# Association of DASH and Depressive Symptoms with BMI over Adulthood in Racially and Socioeconomically Diverse Adults Examined in the HANDLS Study

**DOI:** 10.3390/nu11122934

**Published:** 2019-12-03

**Authors:** Marie Fanelli Kuczmarski, Sharmin Hossain, May A. Beydoun, Ana Maldonando, Michele K. Evans, Alan B. Zonderman

**Affiliations:** 1 Department of Behavioral Health and Nutrition, University of Delaware, 206C McDowell Hall, Newark, DE 19716, USA; 2 Laboratory of Epidemiology and Population Sciences, National Institute on Aging, National Institute of Health, Baltimore, MD 21224, USA; sharmin.hossain@nih.gov (S.H.); baydounm@nih.gov (M.A.B.); ana.maldonado@nih.gov (A.M.); evansm@grc.nia.nih.gov (M.K.E.); zondermana@mail.nih.gov (A.B.Z.)

**Keywords:** DASH diet, depressive symptoms, body mass index, urban adults

## Abstract

Adherence to the Dietary Approaches to Stop Hypertension (DASH) diet is linked to slower weight gain over time. Elevated depressive symptoms may lead to poor quality diets, potentially increasing Body Mass Index (BMI). This study explored these pathways using longitudinal data extracted from 1201–1458 Healthy Aging in Neighborhoods of Diversity across the Life Span (HANDLS) study participants. DASH mean score was computed using four 24 h recalls [visits(v)1 + v2/2: 2004–2013] and depressive symptoms using the Center for Epidemiologic Studies Depression (CES-D) scale (v1 + v2/2: 2004–2013). BMI was measured at v2: 2009–2013 and v3: 2013–2017. Multiple linear mixed regression and mediation modeling were conducted, linking CES-D_(mean)_ and DASH_(mean)_ to BMI [v2 and annualized change ΔBMI (v3-v2)] and exploring mediation of the CES-D_(mean)_–BMI_(v3)_ and DASH_(mean)_–BMI_(v3)_ associations through DASH_(mean)_ and CES-D_(mean)_, respectively. Models were further stratified by sex, race and poverty status. Inverse cross-sectional and longitudinal relationships of DASH_(mean)_ with BMI_(v2)_ and ΔBMI were detected, mainly in women and <125% of poverty participants. CES-D_(mean)_ was not associated with BMI_(v3)_; no mediation was detected through DASH_(mean)_ in all socio-demographic strata. Moreover, the inverse DASH_(mean)_–BMI_(v3)_ total effect was not mediated through CES-D_(mean)_. Future studies should explore other pathways aside from depressive symptoms by which DASH can have a preventive effect on weight status over time.

## 1. Introduction

Diet quality is associated with body mass index (BMI), waist circumference, and body weight [1,2,3,4,5]. The Dietary Approaches to Stop Hypertension (DASH) diet, characterized by high intakes of fruits, vegetables, low-fat dairy, and whole grains, is recognized as a healthful dietary pattern [6]. Adherence to this high-quality diet is associated with less weight gain with aging [3], as well as weight loss and maintenance [7,8]. In African American women across the US, adherence to the DASH diet was associated with reduced obesity risk over a 16 years follow-up. These women were 21–39 years with normal weight at baseline [9]. Additionally, improvement in adherence to the DASH diet is associated with decreases in BMI and weight, especially in persons at high genetic risk for obesity [10]. 

Among adults in the United States (US), the average weight gain is 1.1 to 2.2 lbs. per year from early to middle adulthood [11]. These modest increases in weight from early adulthood could result in overweight or obesity later in life. Zheng and colleagues have reported that weight gain in early adulthood to age 55 years is associated with increased risk of developing chronic conditions such as type 2 diabetes, hypertension, and cardiovascular disease [11]. 

A systematic review and meta-analyses of data from longitudinal studies conducted by Luppino and colleagues confirmed the reciprocal link between depression and obesity [12]. Obesity increased the risk of depression and depression predicted developing obesity. Like obesity, depression increases the risk of heart disease, diabetes, and stroke [13,14]. Using two longitudinal and 44 cross-sectional studies, Preiss and colleagues identified potential biopsychosocial factors associated with the bi-directional obesity–depression relationship [15]. Nine of the 44 studies explored eating behaviors. However, none examined diet quality. A prospective study of 14,051 individuals with a median follow-up period of 8 years found that moderate adherence to the DASH diet was associated with a lower risk of depression. The association was U shaped [16]. 

Low diet quality, assessed by Healthy Eating Index-2005, was associated with elevated BMI and depression in a sample of low-income African Americans residing in “food deserts” in Pittsburgh, Pennsylvania, US [17]. To our knowledge, the association of the DASH diet and depressive symptoms with BMI has not been examined longitudinally. The objective of this study was to explore cross-sectional and longitudinal associations among depressive symptoms, diet quality (DASH score), and BMI, adjusting for demographic and lifestyle factors.

## 2. Methods

### 2.1. Background on Healthy Aging in Neighborhoods of Diversity across the Life Span (HANDLS) Study 

The National Institute on Aging, National Institute of Health initiated the HANDLS study in 2004. This 20 year study was designed to examine the relationships between race (African American [AA] and White [W]) and socioeconomic status on health disparities in urban adults. The sample resided in Baltimore City, US. A detailed description of the study and complete listing of HANDLS variables by wave can be found elsewhere [18,19]. Currently, 3 in-person examination waves have been completed, namely baseline wave (visit [v]1: 2004–2009), a first follow-up visit (v2: 2009–2013) and a second follow-up visit (v3: 2013–2017). 

Approval of the study protocol was granted by human Institutional Review Boards at MedStar Health Research Institute, the National Institutes of Environmental Health Sciences, National Institutes of Health, and the University of Delaware. Each participant provided written informed consent after reviewing a protocol booklet and watching a video describing all procedures. Participants received monetary compensation.

### 2.2. Study Sample

**Figure 1:**
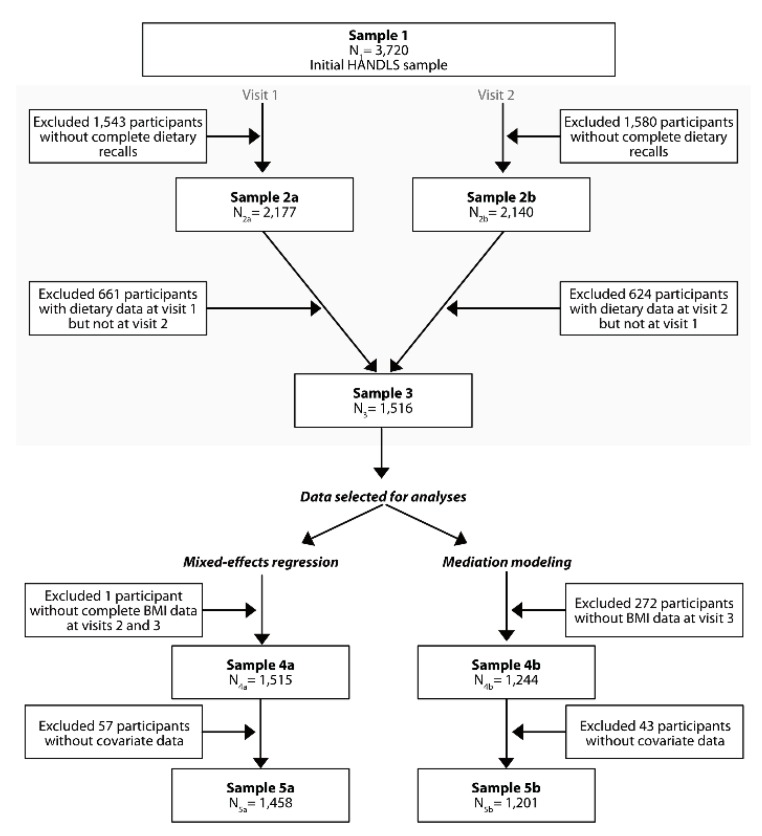
Chart of Subject Selection.

Of the original 3720 HANDLS participants recruited at v1 (2004–2009), 2177 completed two 24 h dietary recalls collected at both Phase 1 (household visit), and Phase 2 (MRV visit). At visit 2 (2009–2013), N = 2140 completed two 24 h dietary recalls, with a sub-sample of N = 1516 having dietary data at both v1 and v2. From these dietary data, the DASH diet score was estimated taking the mean between v1 and v2. No further exclusions were made for mixed-effects regression models with BMI as the outcome (i.e., complete at v2 and v3), except for one participant with incomplete data on BMI for both v2 and v3, resulting in a sample of N = 1515. For mediation models with BMI at visit 3 as the outcome of interest, data were available on N = 1241 participants. Accounting for missing data on covariates and on CES-D scores at v1 and v2, the final analytic sample for mediation models was N = 1201, while for mixed models, it consisted of 1458 participants, with an average of 1.8 observations/participant (Figure 1). 

### 2.3. Dietary Methods and Quality

For visits 1 and 2, 24 h recalls were collected using the computerized Automated Multiple-Pass Method (AMPM) developed by the United States Department of Agriculture (USDA) [20,21]. Participants had access to an illustrated food model booklet, as well as cups, spoons, and a ruler, to assist them in estimating accurate quantities of foods and beverages consumed. All interviewers completed a 3 day training workshop and periodic refresher training sessions. Each food and beverage consumed was assigned an 8 digit code from the Food and Nutrient Database for Dietary Studies, matching the date of the wave to the most appropriate database [22]. The codes were assigned by both the USDA Survey Net data processing system and trained dietary coders. Food descriptions and quantity data collected for each food in the AMPM were imported into Survey Net, a computer-assisted food coding system. Coders viewed this information as they selected food codes and quantities in Survey Net to match the AMPM food intake data [21]. Mean alcohol intake/1000 kcal was estimated from the 24 h recalls. For each food reported in the AMPM, the participant was asked where the food was obtained. Energy from grocery stores was calculated using this feature, summed for everyone per recall day, then averaged over the two recalls per visit, and then averaged across v1 and v2. This variable was included since energy from grocery stores may be directly associated with diet quality.

The score for the DASH diet was determined for each participant using the formula reported by Mellen et al. [23]. The DASH score was calculated using nine nutrients, namely total fat, saturated fat, protein, fiber, cholesterol, calcium, magnesium, sodium, and potassium. Micronutrient goals were targeted by the formula and expressed per 1000 kcal. The total DASH score was generated by summing across all nutrient targets, ranging from 0 to 9. A value 1 was assigned if the participant achieved the DASH target for a nutrient and a value of 0.5 was assigned if the intermediate target was achieved. Zero was assigned if neither target was met [23]. Those estimates were subsequently averaged to obtain the mean DASH total and component scores for both days combined for each of the two visits: DASH_(mean)_ = [DASH_(v1)_ + DASH_(v2)_]/2.

### 2.4. Demographic and Health-Related Measures

Demographic characteristics measured included age (years), sex, race, poverty status, education, and smoking status. Race was self-reported as AA or W. Income was self-reported household income either <125% or >125% of the 2004 Health and Human Services poverty guidelines; hereafter termed “poverty” [24]. Education was categorized as <12th grade education or ≥12th grade education/general equivalency diploma. Literacy was assessed by the reading subtest of the Wide Range Achievement Test, 3rd ed., [WRAT-3] [25]. Cigarette smoking was coded as a current smoker or non-smoker (never or former smoker). Illicit drug use was self-reported and categorized as either current drug user of marijuana, cocaine, and/or opiates or never/former drug user. Current drug use had a time frame of 6 months or less. Self-reported health was categorized as based on the participants’ response to the question, “In general, would you say your health is: Excellent, Very good, Good, Fair, or Poor?”. Employment status was based on response by participant as a yes or no to the question “Were you employed in the past month?”

### 2.5. Depressive Symptoms

The Center for Epidemiologic Studies Depression Scale (CES-D), a 20 item self-report symptom rating scale that assesses affective and depressed mood, was used to measure depression [26]. A score ≥16 on the CES-D is associated with clinical depression based on the Diagnostic and Statistical Manual of Mental Disorders, 5th edition, criteria [27,28]. Those estimates were based on the mean CES-D total scores combined for each of the two visits: CES-D_(mean)_ = [CES-D_(v1)_ + CES-D_(v2)_]/2.

### 2.6. Outcome Measure

BMI was calculated as the ratio of weight (kg) to height (m) squared. Weight was obtained using a calibrated Med-weigh, model 2500 digital scale, and height was measured with the participant’s heels and back against a height meter (Novel Products, Inc., Rockton IL, USA). BMI at v2 and v3 and annual rates of change between v2 and v3 were used in linear mixed-effects regression models. 

### 2.7. Data Handling and Statistical Analysis

Stata Release 16.0 [29] was used to complete all statistical analyses. First, study sample characteristics were assessed by tertiles of mean CES-D tertiles. To test a linear trend relationship between CES-D tertiles and continuous characteristics, a bivariate ordinary least square (OLS) regression was used with CES-D entered as an ordinal predictor of each continuous variable of interest. Associations between categorical study characteristics and CES-D tertiles were evaluated with χ^2^ tests. Second, linear regression models were conducted to test associations between CES-D_(mean)_ and DASH score total scores across socio-demographic groups, with two models presented: Model 1 [adjusted for total energy intake: (kcalv1 + kcalv2)/2]; Model 2 [adjusted for total energy intake, age, sex, race, poverty status, education, literacy, employment, smoking, drug use, self-reported health, mean energy intake, and mean energy intake from grocery stores]. Third, multiple linear mixed-effects regression models were conducted to test associations between CES-D_(mean)_, DASH_(mean)_ and longitudinal change in the BMI between first (v2) and second follow-up (v3), adjusting for baseline (v1) and fixed characteristics [v1 age, v2 age, sex, race, poverty status, education, literacy, employment status, current smoking status, current drug use, self-rated health, mean of total energy intake, and mean energy intake from grocery stores]. Each model included TIME (set at zero for v2 BMI, and time elapsed to v3 for second follow-up BMI) and interaction terms between TIME and key exposures (CES-D_(mean)_ and DASH_(mean)_ scores) as well as covariates. Those interaction terms are interpreted as the effects of exposures and covariates on the slope or annual rate of change in the BMI (between 2009–2013 and 2013–2018). The main effects of exposures and covariates were also included in each model and are interpreted as fixed effects of exposures on outcome, in this case, BMI at v2. Repeated outcome measures ranged between 1 and 2, with a mean of 1.8 visits per participant. We assumed the unavailability of outcomes to be missing at random. In the overall sample, three models were run: Model 1 (Both CES-D and DASH), Model 2 (CES-D alone) and Model 3 (DASH alone). In addition to the overall models, mixed-effects regression models were stratified separately by sex, race and poverty status. Heterogeneity in the effect of exposure on change in outcome was formally tested by adding two-way and three-way interaction terms between sex/race/poverty status, TIME and exposure (CES-D/DASH). A separate sensitivity analysis was performed with mean alcohol intake/1000kcal from v1 and v2. 

BMI at second follow-up (i.e., v3) was considered as an endogenous variable that was potentially associated with both CES-D_(mean)_ and DASH_(mean)_. To test mediation, two methods were used. First, mediation models were carried out where CES-D_(mean)_, socio-demographic, lifestyle and health-related factors [similar to those used in the mixed-effects model] were exogenous to DASH(mean) and BMI using z-scores for each. Second, when relaxing the assumption of additivity (RAA) between zCES-D_(mean)_ and the zDASH_(mean)_ score by including an interaction term, we further computed four estimates with their standard errors (SEE) and *p*-values, namely the controlled direct effect (CDE), the natural direct effect (NDE), the natural indirect effect (NIE) and the marginal total effect (MTE). In another set of models, zDASH_(mean)_ was the exposure while zCES-D_(mean)_ was the mediator. This approach is detailed elsewhere [30]. For example, when DASH_(mean)_ is the mediator in the mediation model, the CDE is the effect of fixing X [i.e., zCES-D_(mean)_] to 1 versus 0 (i.e., 1 SD higher than the mean vs. the mean) while controlling M (DASH_(mean)_) to some defined reference value m. In this case, M is set at a value close to the mean, namely zero. The NDE is the same setting of the exposure X, but this time M (zCES-D_(mean)_) is fixed not to a single predefined value m, but instead, a value that is theoretically distinctive for each data-point in the analytical sample. It is the value that M would have taken at the referent value of the exposure (in this case, the exposure level that is at the mean). The NIE is the outcome contrast observed when holding exposure X [i.e., CES-D_(mean)_] constant at its mean, and contrasting two different M [DASH(mean)] values: the value of the DASH_(mean)_ score that would be observed for that person under the X value [CES-D_(mean)_] of the population mean and the value of DASH_(mean)_ that would be observed for that person under the 1 SD higher X value. The total effect is the sum of the NIE and the NDE. It is the total effect of varying X by 1 SD, irrespective of M (or the DASH score) [30]. All analyses were conducted overall and stratified separately by sex, race, and poverty status. 

The non-random selection of participants with complete data from the target study population can often lead to selection bias. To account for this type of bias, a 2-stage Heckman selection model was constructed [31], using a probit model to obtain an inverse Mills ratio at the first stage (derived from the predicted probability of being selected out of the sample with complete 24 h recalls at baseline (N = 2177, see Figure 1), conditional on the covariates in the probit model, mainly baseline age, sex, race, poverty status and education), as was done in earlier studies [32]. Specifically, participants included the final analytic sample used for the mixed-effects linear regression models (*n* = 1458) differed from the remaining sample of out of the initial N = 3720 by having a smaller proportion male (41% vs. 48%, *p* < 0.05). Similar patterns of socio-demographic differences were noted when comparing the final analytic sample used for mediation models to the excluded group from the initial sample (*n* = 3720). 

Type I error was set at 0.05 for main effects and 0.10 for interaction terms due to the latter’s reduced statistical power compared to the former.

## 3. Results

### 3.1. Characteristics of Study Participants by CES-D Tertiles 

Crude sample characteristic distribution by tertiles of mean CES-D between v1 and v2 are provided in Table 1. Women were more likely than men to be in the uppermost CES-D tertile (*p* = 0.004). Higher CES-D was also linked to lower SES assessed by poverty status, lower education and literacy levels, and unemployment status. Higher CES-D was also directly related to tobacco use and poorer self-rated health. Overall, no association was detected between CES-D_(mean)_ tertiles and BMI measured at either visit (or mean). However, the DASH diet (v1 and mean) was inversely associated with CES-D.

### 3.2. DASH Diet Score by CES-D Tertiles: Energy-Adjusted and Full Model

The examination of the stratum-specific association between CES-D and DASH total score (means: v1/v2) found a direct linear dose-response relationship after adjustment for mean energy intake (Model 1) as well as age, sex, race, poverty status, education, literacy, employment status, smoking, drug use, self-reported health, mean energy intake and mean energy from grocery stores (Model 2) (Table 2). The results of Model 1 detected a negative association between CES-D and total DASH score overall (*p* = 0.001), and most strata with the exception of African Americans and individuals living below poverty. The fully adjusted model, namely Model 2, demonstrated that this negative association was restricted to individuals living above poverty (*p* = 0.024). Except for poverty status, CES-D did not interact with socio-demographic factors in its association with DASH(mean) total score in Model 2. 

### 3.3. DASH Diet Score, CES-D, and BMI Over Time: Mixed-Effects Regression Models

The mixed models provided in Table 3 depicted the cross-sectional and longitudinal associations of CES-D and DASH total score (mean, v1/v2) with BMI. Overall, a better dietary quality (higher DASH score) was associated with a lower BMI (v2), after adjusting for all potential confounders. The total effect of CES-D (Model 2) was not detectable, while the DASH diet score was inversely related to BMI (Model 3) overall, in women, White participants and in “below poverty” groups. The results were indicative of a direct protective effect of the DASH diet on BMI over time. Heterogeneity by sex, race and poverty status was found for both cross-sectional and longitudinal associations of CES-D and DASH with BMI. The separate sensitivity analyses that further adjusted for alcoholic beverages per 1000 kcal showed no confounding effect by this additional covariate. 

### 3.4. DASH Diet Score as a Mediator between CES-D and BMI at Last Visit: RAA Mediation Models

A mediation analysis (Table 4), yielded no significant associations. Therefore, it was apparent that DASH_(mean)_ did not mediate the relationship between CES-D_(mean)_ and BMI_(v3)_, even after relaxing the additivity assumption. 

### 3.5. CES-D Score as a Mediator between DASH and BMI at Last Visit: RAA Mediation Model

In mediation models whereby the CES-D score was allowed to mediate the total association between DASH and BMI, only the direct and total effects (DASH→BMI) were statistically significant, suggestive of an inverse association, while the indirect effect (DASH→CES-D→BMI) was not (Table 4). This finding implies that another pathway that is independent of CES-D may be explaining the potential protective effect of DASH against adiposity measured with BMI.

## 4. Discussion

This study examined the association between depressive symptoms assessed by the CES-D scale and diet quality assessed by DASH score with BMI using longitudinal and mediation analyses while stratifying by key socio-demographic factors. Results of multiple mixed-effects linear regression models indicated an inverse cross-sectional relationship between DASH_(mean)_ and BMI_(mean)_, mainly in women and participants with incomes <125% of poverty. Previous research found that the food intake of this sample tended to resemble the Western diet, with low adherence to the DASH diet [33]. However, the protective effects of the DASH dietary pattern from an increase in BMI with age were observed in the HANDLS study participants. Similarly, in this sample an inverse relationship between DASH score and 10 year atherosclerotic cardiovascular risk has also been reported [33].

Nevertheless, no significant longitudinal associations were detected between CES-D_(mean)_ and annualized BMI change. Moreover, CES-D_(mean)_ was not associated with BMI_(v3)_ and no mediation was detected through DASH_(mean)_ in all socio-demographic strata. The lack of association might be attributed to the fact that the mean BMI at baseline of this sample was 30 kg/m^2^, indicating that most participants were either overweight or obese.

### 4.1. The Link between DASH Score and Depressive Symptoms

The association between diet quality indices and depressive symptoms has been explored only recently in longitudinal studies of middle-aged adults [16,34,35]. Averaging both DASH and CES-D scores over two study visits (2004–2013), our study found a negative association between diet quality and depressive symptoms in the HANDLS study sample after adjusting for energy and several socio-economic factors. This finding was consistent with that reported by researchers who performed a systematic review and meta-analyses of observational studies [34,35]. Perez-Cornago and colleagues reported that the Fung DASH score had a significant, inverse non-linear relationship with depression incidence eight years later. The Mellen DASH score was weaker in magnitude than that found for Fung DASH score [16]. However, a review of studies examining the link between DASH and depression in adults found either no or positive significant associations (either cross-sectionally or longitudinally) between these two factors [35,36]. These inconsistent findings may be due to the differences in scoring systems for DASH scores and to the covariates included in statistical models. However, a meta-analysis of five longitudinal studies found that baseline DASH scores were inversely predictive of change in depressive symptoms 5–6 years later, such that higher DASH diet scores led to greater decreases in depressive symptoms [34]. It seems apparent that longitudinal research on the association between the DASH diet and depressive symptoms is mixed and requires further examination.

### 4.2. The Link between DASH Score and BMI

The study findings are consistent with cross-sectional studies across the world that have provided evidence of the association of higher DASH scores with lower BMI [8,32,37,38,39,40,41]. Our longitudinal analyses also provided evidence of an inverse association. However, previous research has focused on examining the relationship between DASH diet scores and BMI and obesity [42,43,44] has not always found this association. For instance, in one of the largest longitudinal studies among young and middle-aged women (*n* = 19,885, 21–39 years at baseline), higher diet scores (both DASH and Alternate Healthy Eating Index [AHEI]) at baseline were negatively associated with obesity incidence at 6 year follow-up (but only for women that had a normal BMI at baseline and after controlling for age, total energy intake and BMI). These associations became non-significant after further controlling for vigorous exercise, television watching, education, geographic region, smoking status, parity and age at first birth. There were no significant associations between diet scores and obesity incidence for women who were overweight at baseline [9]. As mentioned previously, differing scoring systems for DASH and covariates may contribute to inconsistency of results. In another large cross-sectional study (N = 2767 men and women, 20–90 years), individuals <45 years with higher DASH scores were less likely to be individuals who were normal weight with susceptible cardiovascular abnormalities, a group known as metabolically obese normal weight (MONW), compared to those with lower DASH scores after controlling for all covariates. However, there were no significant associations between DASH diet scores and MONW for those who were older (i.e., ≥45 years for men and postmenopausal women) [45].

### 4.3. The Link between Depressive Symptoms and BMI

The findings of this study found no association with depressive symptoms and BMI over time. However, research by Beydoun and colleagues reported an association between baseline depressive symptoms and central adiposity among HANDLS study participants. Even though marginally significant, the association was among two mechanisms mediating the SES disparity in central adiposity [46]. Moreover, baseline central adiposity was also directly linked to follow-up depressive symptoms, specifically among African-American women (*p* < 0.10) [46]. In fact, Beydoun and colleagues found two closely fitting pathways indicating that there were bi-directional relationships between diet quality (measured with the 2010 Healthy Eating Index), depressive symptoms and central adiposity [46].

In contrast, one of the two temporal directions were present in other Baltimore cohort studies. For instance, Sutin and colleagues found that women with depressed affect at some point in their lives had greater increases in BMI, waist and hip circumference across the adult lifespan. They used data on 2251 adults residing in Baltimore (mean age 57.9 years). However, baseline adiposity showed no association with depressive symptom trajectory in both men and women [47]. Another multiethnic study from Baltimore city, (men and women, 30–89 years at baseline, *n* = 1071), showed baseline depression predicted weight gain during an 11 year follow up [48]. Several other studies found associations, between baseline obesity (or adiposity) and follow-up depression across multiple socio-demographic groups [49,50,51].

Moreover, data from the Nurse’s Health Study revealed that baseline depression predicted increased obesity risk at the 10 year follow-up, and vice-versa at follow-up [52]. These women (*n* = 65,955) were racially diverse and between 54 and 79 years. A similar, bi-directional relationship in a slightly younger age group (45–50 years, follow-up:12 years) was observed by Singh and colleagues in women, whereby weight gain was positively associated with increased prevalence and incidence of depression and both had increased risk of weight gain when used as predictors [53].

### 4.4. Strengths, Limitations and Future Directions

Our study has many notable strengths, including its use of the DASH diet score in relation to CES-D and BMI using repeated measures over time, thus ascertaining the temporality of relationships. Specifically, annual rates of change in BMI (between v2 and v3), as well as BMI at follow-up (v3), was tested against cumulative exposures over two visits of data (v1 and v2), by taking their respective averages. Furthermore, it is among the few studies that tested those associations across race, sex, and income groups. Though our study setting was Baltimore city, our findings can be generalized to comparable populations across the United States, mainly 14 cities of similar population densities and racial distributions [19].

Nevertheless, there were study limitations. The use of USDA AMPM has been shown to reduce bias in the collection of energy intakes [20]; however, there are inherent errors associated with the 24 h dietary recall [54]. The selection of the diet quality method of measurement may have affected some of our key findings. DASH scores were based on only food and beverage intakes. The exclusion of nutritional supplements may be viewed by some as a limitation. Data on supplement usage were only collected at v2 and v3 and revealed that approximately 40% of the sample took supplements. Incorporation of the nutrient intakes from supplement users would increase their DASH scores. Physical activity was not measured at all three visits and therefore it was not possible to assess effect modification by activity in our analyses. Thus, our findings should be interpreted with caution. More longitudinal studies are needed to replicate our findings in comparable samples of urban adults.

## 5. Conclusions

In this group of community-dwelling urban adults, an energy-adjusted increase in the DASH diet score had an inverse impact on BMI over time. This association was observed more strongly among Whites, women, and participants with incomes less than 125% of the poverty level. This latter finding highlights the importance of overcoming economic barriers to improve dietary quality, which in turn could improve health outcomes. The lack of a mediated effect between CES-D and BMI through DASH score (or a mediated effect between DASH and BMI through CES-D) highlights the need to investigate this pathway further as well as to conduct future randomized trials while accounting for economic barriers.

## Figures and Tables

**Table 1:** Study sample characteristics by tertile of depressive symptoms [CES-D score (mean)^1^], HANDLS 2004–2013.

Characteristics	Depressive Symptoms (Mean) Tertiles ^1^			
	T_1_	T_2_	T_3_	*p* ^2^
	(N = 519)	(N = 473)	(N = 466)	
**Demographic and Socio-economic**				
Sex at V1, % male	42.4	44.6	34.7	0.004
Age at V1, years (X ± SE)	49.0 ± 0.4	48.0 ± 0.4	48.1 ± 0.4	0.15
Age at V2, years (X ± SE)	53.7 ± 0.4	52.7 ± 0.4	52.9 ± 0.4	0.18
Age at V3, years (X ± SE)	57.9 ± 0.4	56.5 ± 0.5	56.4 ± 0.4	0.022
African American at V 1, %	60.3	61.3	59.2	0.808
Poverty status at V1, % (<125% poverty)	31.4	39.1	53.2	<0.0001
Education at V1, years Completed, %				
<High School	3.7	6.1	10.0	<0.0001
High School	45.7	62.8	65.4	
>High School	50.7	31.1	24.7	
Literacy at V1, WRAT-3 score				<0.0001
<36, %	11.4	22.0	29.2	
37–40, %	13.7	16.3	17.2	
41–46, %	27.9	29.2	28.4	
≥47, %	47.0	32.6	25.3	
Unemployed in last month at V1, % yes	23.3	31.3	45.3	<0.0001
Unemployment in last month, % missing	17.0	17.8	19.1	
**Health-Related**				
Self-rated health at V1				<0.0001
Poor/Average, %	11.9	19.2	39.7	
Good, %	39.3	44.6	39.5	
Very good/Excellent %	48.7	36.2	20.8	
CES-D score (mean of V1& V2) (X ± SE)	5.15 ± 0.11	13.59 ± 0.12	26.85 ± 0.33	<0.0001
Drug and tobacco use at V1				
Any drug, current user, % ^3^	43.4	46.3	48.1	0.306
Any drug, missing, %	8.3	6.6	9.0	
Tobacco, current user, %	28.7	45.2	49.6	<0.0001
Tobacco, missing, %	10.2	7.4	11.2	
BMI at V2, kg/m^2^ (X ± SE)	30.6 ± 0.3	30.2 ± 0.4	30.8 ± 0.4	0.542
BMI at V3, kg/m^2^ (X ± SE)	31.1 ± 0.4	30.2 ± 0.4	31.0 ± 0.4	0742
BMI (mean of V2 & vV3), kg/m^2^ (X ± SE)	31.0 ± 0.3	30.7 ± 0.4	30.9 ± 0.4	0.671
BMI annual rate of change (ΔBMI), (X ± SE) ^4^	0.01 ± 0.04	0.056 ± 0.04	0.08 ± 0.05	0.596
**Diet-Related**				
DASH total score at V1 (X ± SE)	1.89 ± 0.07	1.62 ± 0.06	1.64 ± 0.06	0.0020
DASH total score at V2 (X ± SE)	1.83 ± 0.06	1.69 ± 0.06	1.76 ± 0.06	0.211
DASH total score (mean) (X ± SE) ^5^	1.86 ± 0.05	1.65 ± 0.05	1.70 ± 0.05	0.0046
Total Energy intake at V1, kcal/d (X ± SE)	2039 ± 43	2023 ± 40	1941 ± 44	0.218
Total Energy intake at V2, kcal/d (X ± SE)	2118 ± 39	2079 ± 39	1950 ± 37	0.0060
Total Energy intake (mean), kcal/d (X ± SE) ^5^	2079 ± 36	2051 ± 34	1945 ± 33	0.018
Energy Intake from Grocery Store				
Energy intake at V1, kcal/d (X ± SE)	1526 ± 38	1552 ± 38	1535 ± 41	0.893
Energy intake at V2, kcal/d (X ± SE)	1551 ± 36	1608 ± 37	1536 ± 35	0.333
Energy intake (mean), kcal/d (X ± SE) ^5^	1539 ± 32	1580 ± 32	1535 ± 31	0.546

Abbreviations: DASH = Dietary Approaches to Stop Hypertension; HANDLS = Healthy Aging in Neighborhood of Diversity across the Lifespan; SE = Standard Error; T = tertile; V = visit; WRAT-3 = Wide Range Achievement Test, 3rd revision. ^1^ The Center for Epidemiologic Studies Depression (CES-D) scale is used to measure depression using a 20 item questionnaire. The scores range from 0–60 and a higher score generally indicates more symptomatology. Measured as mean across V1 and V2. ^2^ *p*-value from one-way ANOVA (continuous variables) or from χ^2^ test (categorical variables). Post-hoc Bonferroni corrected t-test for the null hypothesis of no between tertile differences, taking T_1_ as the referent. ^3^ Drugs include marijuana, cocaine, and heroin. ^4^ ΔBMI = rate of change between V2 and V3. ^5^ Measured as mean across V1 and V2.

**Table 2:** CESD-D tertiles as predictors of DASH (mean) total score, stratifying by sex, race and poverty status: multiple ordinary least square regression models, HANDLS 2004–2013.

		Model 1: CES-D (Mean) Tertiles ^1^			Model 2: CES-D (Mean) Tertiles ^1^		
		β ± SE (T_2_ vs. T_1_)	β ± SE (T_3_ vs. T_1_)	*p*-Trend ^2^	β ± SE (T_2_ vs. T_1_)	β ± SE (T_3_ vs. T_1_)	*p*-Trend ^2^
**DASH (mean) total score**							
Overall	N = 1458	−0.22 ± 0.06	−0.21 ± 0.06	0.001	−0.15 ± 0.06	−0.10 ± 0.07	0.116
Men	N = 592	−0.18 ± 0.09	−0.23 ± 0.10	0.013	−0.12 ± 0.09	−0.12 ± 0.10	0.188
Women	N = 866	−0.25 ± 0.09	−0.20 ± 0.09	0.023	−0.21 ± 0.09	−0.11 ± 0.09	0.224
Whites	N = 579	−0.27 ± 0.11	−0.32 ± 0.11	0.004	−0.17 ± 0.11	−0.13 ± 0.12	0.263
AA	N = 879	−0.17 ± 0.07	−0.14 ± 0.08	0.057	−0.16 ± 0.08	−0.10 ± 0.08	0.189
Above poverty	N = 862	−0.24 ± 0.08	−0.29 ± 0.09	0.001	−0.16 ± 0.08	−0.19 ± 0.09	0.024
Below poverty	N = 596	−0.13 ± 0.10	−0.01 ± 0.10	0.768	−0.14 ± 0.10	+0.02 ± 0.10	0.706

Abbreviations: CES-D = The Center for Epidemiologic Studies Depression scale; DASH = Dietary Approaches to Stop Hypertension; HANDLS = Healthy Aging in Neighborhood of Diversity across the Lifespan; OLS = Ordinary Least Square; SE = Standard Error. ^1^ Values are regression coefficients and their standard errors (β ± SE) from an OLS linear regression model with Y = mean DASH total score (v1/v2) and the key predictor being tertile of mean CES-D score (v1/v2), contrasting the middle tertile with the lowest tertile (T_2_ vs. T_1_) and the uppermost tertile with the lowest tertile (T_3_ vs. T_1_). Model 1 is adjusted for energy intake (mean of visits 1 and 2), while Model 2 is further adjusted for age, sex, race, poverty status, education, literacy, employment, smoking, drug use, self-reported health, mean energy intake, and mean energy intake from grocery stores. ^2^ *p*-trend was derived from a model similar to Model 1, but with the key predictor CES-D tertiles entered as a single ordinal variable rather than two dummy variables. *p* values for null hypothesis that β = 0 (i.e., T_2_ vs. T_1_ and/or T_3_ vs. T_1_).

**Table 3:** CES-D_(mean)_ and DASH_(mean)_ total score as predictors of first follow-up and rate of change in BMI, overall and stratifying by sex, race and poverty status: multiple linear mixed-effects regression models, HANDLS 2004–2018 ^1^.

	Overall	Men	Women	Whites	African Americans	Below Poverty	Above Poverty
**BMI**	*(n = 1458; k = 1.8)*	*(n = 592; k = 1.8)*	*(n = 866; k = 1.8)*	*(n = 579; k = 1.8)*	*(n = 879; k = 1.8)*	*(n = 596; k = 1.8)*	*(n = 862; k = 1.8)*
**Model 1:**							
TIME	+0.32 ± 0.15 *	+0.29 ± 0.20	+0.51 ± 0.20 *	+0.42 ± 0.18	+0.28 ± 0.39	+0.38 ± 0.23	+0.36 ± 0.34
CES-D_(mean)_	+0.01 ± 0.02	−0.01 ± 0.03	+0.02 ± 0.03	−0.02 ± 0.03	+0.03 ± 0.03	−0.01 ± 0.03	+0.02 ± 0.03
CES-D_(mean)_ × TIME	+0.001 ± 0.003	−0.007 ± 0.004	+0.004 ± 0.003	+0.002 ± 0.003	+0.001 ± 0.003	+0.005 ± 0.004	−0.004 ± 0.003
DASH_(mean)_	−0.55 ± 0.20 **^,2^	−0.13 ± 0.29	−0.75 ± 0.27 **^,2^	−0.94 ± 0.28 **^,2^	−0.05 ± 0.27	−0.96 ± 0.34 **^,2^	−0.27 ± 0.24
DASH_(mean)_ × TIME	−0.04 ± 0.02	−0.02 ± 0.03	−0.04 ± 0.03	−0.04 ± 0.03	−0.05 ± 0.03	−0.09 ± 0.04 *^,2^	−0.02 ± 0.02
**Model 2:**							
TIME	+0.31 ± 0.15 *	+0.29 ± 0.20	+0.50 ± 0.20 *	+0.42 ± 0.18 *	+0.30 ± 0.39	+0.38 ± 0.23	+0.37 ± 0.34
CES-D_(mean)_	+0.01 ± 0.02	−0.01 ± 0.03 ^2^	+0.02 ± 0.03	−0.02 ± 0.03	+0.03 ± 0.03	−0.01 ± 0.03	+0.02 ± 0.03
CES-D _(mean)_ × TIME	+0.001 ± 0.003	−0.007 ± 0.004 ^2^	+0.005 ± 0.003	+0.003 ± 0.003	+0.001 ± 0.003	+0.005 ± 0.004 ^2^	−0.005 ± 0.003
**Model 3:**							
TIME	+0.32 ± 0.15 *	+0.22 ± 0.20	+0.56 ± 0.19 **	+0.45 ± 0.18 *	+0.29 ± 0.39	+0.44 ± 0.22	+0.32 ± 0.34
DASH_(mean)_	−0.55 ± 0.20 **^,2^	−0.13 ± 0.29	−0.76 ± 0.27 **^,2^	−0.93 ± 0.28 **^,2^	−0.05 ± 0.27	−0.96 ± 0.34 **	−0.28 ± 0.24
DASH_(mean)_ × TIME	−0.04 ± 0.02	−0.02 ± 0.04	−0.04 ± 0.03	−0.04 ± 0.03	−0.05 ± 0.03	−0.09 ± 0.04 *	−0.02 ± 0.02

Abbreviations: DASH = Dietary Approaches to Stop Hypertension; HANDLS = Healthy Aging in Neighborhood of Diversity across the Lifespan; SE = Standard Error; CES-D = The Center for Epidemiologic Studies Depression scale. Note: * *p* < 0.05; ** *p* < 0.01. ^1^ Values are fixed effects regression coefficients from mixed-effects linear regression models (γ ± SE). Models were adjusted for visit 1 age, visit 2 age, sex, race, poverty status, educational attainment, literacy, employment status, current smoking status, current drug use, self-rated health, mean of total energy intake, and mean energy intake from grocery stores. ^2^ *p* < 0.05 for null hypothesis of no difference by sex, race, or poverty status based on two-way and three-way interaction terms with CES-D/DASH and TIME. First-visit age was centered at 48. Second-visit age was centered at 53. Energy intake was centered at 2027 kcal/d. Energy from stores centered at 1551 kcal/d. DASH_(mean)_ was centered at 1.74. CES-D_(mean)_ centered at 14.8. *p* < 0.05 for the null hypothesis of no difference by sex, race, or poverty status based on two-way and three-way interaction terms with CES-D/DASH and TIME.

**Table 4:** zCES-D_(mean)_, zDASH_(mean)_ total score and zBMI_(v3)_ at second follow-up: mediation model relaxing the assumption of no interaction between exposure and mediator and stratifying by sex, race and poverty status, HANDLS 2004–2018 ^1^.

	Controlled Direct Effect			Natural Direct Effect			Natural Indirect Effect			Marginal Total Effect		
	*β*	(SEE)	*P_wald_*	*β*	(SEE)	*P_wald_*	*β*	(SEE)	*P_wald_*	*β*	(SEE)	*P_wald_*
**CES-D→DASH→BMI ^2^**												
Overall sample, *n* = 1201	−0.007	0.030	0.82	−0.007	0.030	0.82	+0.003	0.005	0.59	−0.004	0.030	0.89
Men, *n* = 469	−0.060	0.050	0.22	−0.060	0.050	0.18	+0.001	0.003	0.76	−0.060	0.050	0.19
Women, *n* = 732	+0.030	0.040	0.46	+0.024	0.042	0.57	+0.003	0.008	0.72	+0.027	0.042	0.53
Whites, *n* = 587	−0.010	0.047	0.82	−0.030	0.046	0.52	+0.009	0.012	0.44	−0.020	0.047	0.67
African Americans, *n* = 714	+0.017	0.042	0.68	+0.017	0.042	0.69	+0.001	0.002	0.74	+0.017	0.042	0.68
Below poverty, *n* = 483	+0.013	0.047	0.79	+0.018	0.047	0.70	−0.003	0.008	0.66	+0.014	0.047	0.76
Above poverty, *n* = 718	−0.034	0.043	0.42	−0.035	0.043	0.41	+0.004	0.005	0.35	−0.031	0.043	0.47
**DASH→CES-D→BMI ^3^**												
Overall sample, *n* = 1201	−0.14	0.03	<0.001	−0.14	0.03	<0.001	+0.001	0.001	0.63	−0.14	0.03	<0.001
Men, *n* = 469	−0.05	0.05	0.26	−0.06	0.05	0.22	+0.001	0.002	0.73	−0.06	0.05	0.23
Women, *n* = 732	−0.14	0.04	<0.001	−0.15	0.04	<0.001	+0.001	0.002	0.75	−0.15	0.04	<0.001
Whites, *n* = 587	−0.18	0.04	<0.001	−0.18	0.04	<0.001	+0.003	0.004	0.48	−0.18	0.04	<0.001
African Americans, *n* = 714	−0.03	0.04	0.43	−0.03	0.04	0.43	−0.001	0.002	0.76	−0.04	0.04	0.42
Below poverty, *n* = 483	−0.19	0.05	0.001	−0.19	0.05	<0.0001	−0.001	0.002	0.76	−0.20	0.05	<0.0001
Above poverty, *n* = 718	−0.07	0.04	0.071	−0.07	0.04	0.066	+0.001	0.002	0.48	−0.07	0.04	0.072

Abbreviations: DASH = Dietary Approaches to Stop Hypertension; HANDLS = Healthy Aging in Neighborhood of Diversity across the Lifespan; CES-D = The Center for Epidemiologic Studies Depression scale; OLS = Ordinary Least Square; SEE = Standard Error of the Estimate. ^1^ Multivariate OLS models adjusted for baseline age, sex, race, poverty status, educational attainment, literacy, employment status, current smoking status, current drug use, self-rated health, mean of total energy intake and mean energy intake from grocery store. For CDE, zDASH_(mean)_ was set at a value of zero. Only results with significant total effects at type I error of 0.05 were presented. ^2^ DASH diet as a mediator. ^3^ CES-D as a mediator.
